# Management of severe paediatric malaria in resource-limited settings

**DOI:** 10.1186/s12916-014-0263-6

**Published:** 2015-03-03

**Authors:** Kathryn Maitland

**Affiliations:** Wellcome Trust Centre for Clinical Tropical Medicine, and Department of Paediatrics, Faculty of Medicine, Imperial College, London, UK; Kilifi Clinical Trials Facility, KEMRI-Wellcome Trust Research Programme, Kilifi, Kenya

**Keywords:** Malaria, Africa, Children, Mortality, Clinical Trial, Treatment, Bacterial Infection, Supportive Care

## Abstract

Over 90% of the world’s severe and fatal *Plasmodium falciparum* malaria is estimated to affect young children in sub-Sahara Africa, where it remains a common cause of hospital admission and inpatient mortality. Few children will ever be managed on high dependency or intensive care units and, therefore, rely on simple supportive treatments and parenteral anti-malarials. There has been some progress on defining best practice for antimalarial treatment with the publication of the AQUAMAT trial in 2010, involving 5,425 children at 11 centres across 9 African countries, showing that in artesunate-treated children, the relative risk of death was 22.5% (95% confidence interval (CI) 8.1 to 36.9) lower than in those receiving quinine. Human trials of supportive therapies carried out on the basis of pathophysiology studies, have so far made little progress on reducing mortality; despite appearing to reduce morbidity endpoints, more often than not they have led to an excess of adverse outcomes. This review highlights the spectrum of complications in African children with severe malaria, the therapeutic challenges of managing these in resource-poor settings and examines in-depth the results from clinical trials with a view to identifying the treatment priorities and a future research agenda.

## Background

**Figure 1:**
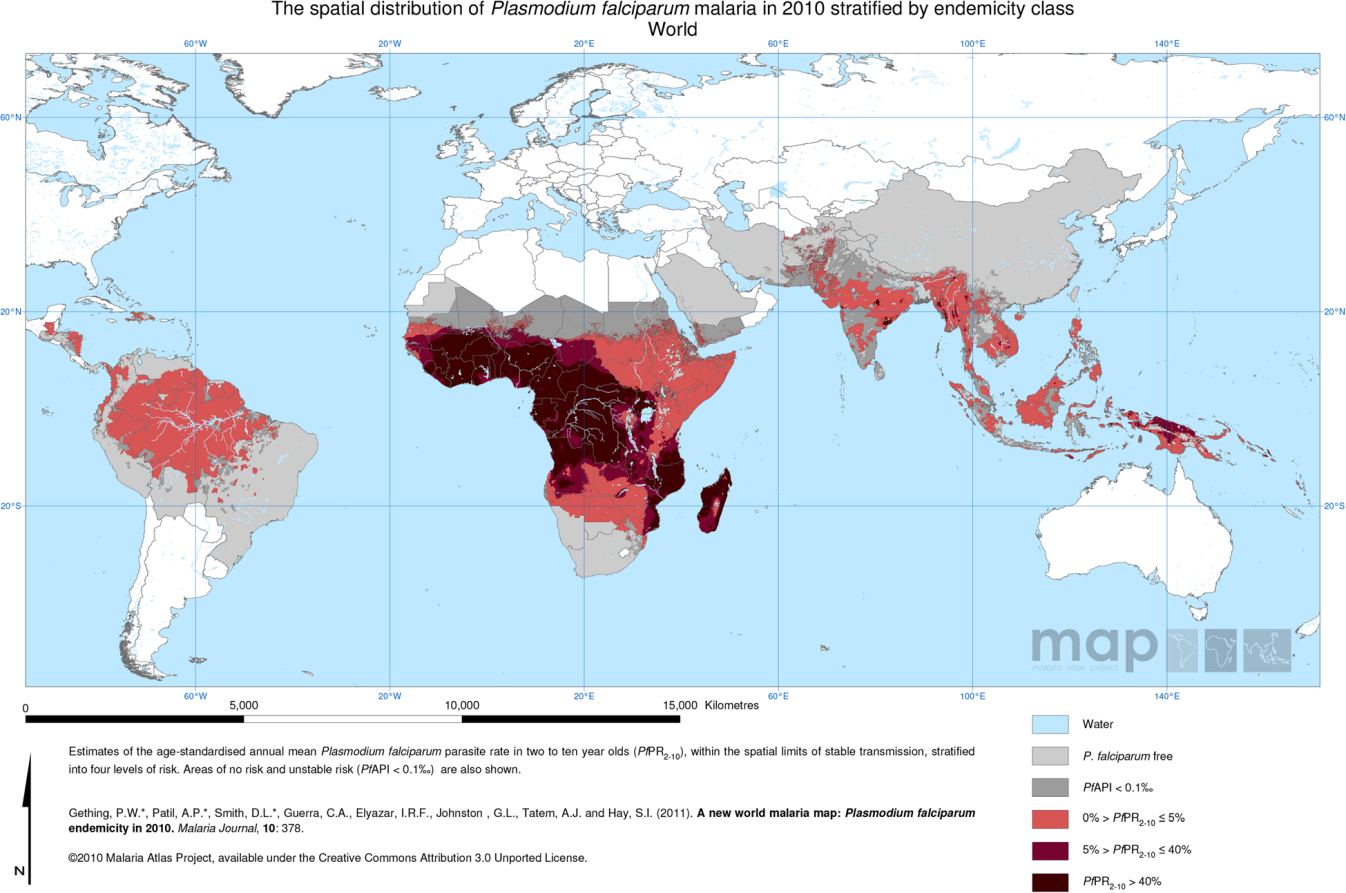
**2010 World Map** ***Plasmodium falciparum*** **parasite rate in 2-** **10 year olds.**

In many parts of the world malaria incidence has declined, in part due to substantial donor investment targeting the scaling up of insecticide-treated nets (ITN) and other vector control measures. Nevertheless, in 2010 it was estimated that there were 500 million episodes of *Plasmodium falciparum* malaria each year [1,2]. In the same year, the World Health Organization (WHO) estimated that there were approximately 650,000 deaths directly attributed to malaria worldwide [3]. The heaviest burden of *P. falciparum* malaria falls on sub-Saharan Africa (sSA), where children under five years old are disproportionately affected by this parasite (Figure 1). Today, 57% of Africa’s populations still live in areas with moderate to high malaria transmission. Ten countries account for 87% of people exposed to the highest malaria endemicities globally, where the *P. falciparum* rates in children two- to ten-years old exceed 50%. Malaria, therefore, remains a very common cause of hospital admission in sSA, and where severe malaria is mainly a disease of children under five years of age. It has been estimated that approximately 90% of the world’s severe and fatal malaria affects young children in sSA [4,5].

Severe malaria is a complex multisystem disorder with a clinical presentation that has many similarities to severe bacterial sepsis, despite distinctive differences in pathogenesis. Children typically present when they are critically ill with life-threatening complications yet, in most African hospitals, few will ever be managed on an intensive care unit or in a high dependency facility. The high, and frequently seasonal, burden of cases of severe malaria presents a unique challenge to health services and clinicians working in resource-limited hospitals. This review intends to provide an overview of the spectrum and complications of severe malaria, highlighting treatment priorities for children managed within low-resource settings in Africa and re-examining what has been learnt from clinical trials to outline a future research agenda. In order to contextualise the current and future relevance of this review and the continued necessity to prioritise research funding for this condition, I will briefly summarise the current status of malaria eradication efforts, aimed at reducing the disease burden and mortality, and accordingly, if successful and sustained, negating the necessity for future investment in this clinical disease.

### Malaria control: are we nearly there yet?

The global strategy towards gradual malaria eradication which has been developed and ratified into policy was reviewed in 2010 in a four-part *Lancet* series [6-9]. It focuses on aggressive control of transmission and investment in vaccine development, insecticides, new treatments and diagnostics. Hospital admissions and deaths due to malaria are, therefore, important barometers for the effectiveness of these measures to control and eliminate malaria. Substantial reductions have been witnessed in several African countries, in some cases directly or plausibly linked to the scaling up of control efforts [10-14]. However, many of the encouraging reports have tended to be from areas with relatively low baseline malaria transmission intensities. In some cases, the greatest decline in malaria hospitalisation preceded the scale-up of ITN use or the introduction of artemisinin combination therapies [13], suggesting that more complex mechanisms are involved. Other sSA countries have documented either no decline in severe or fatal malaria [4], an increase in hospitalisations during the same period [15,16] or a resurgence following sustained control [17]. The downstream effects on the spectrum of severe malaria are uncertain, although there are reports that cerebral malaria is now being witnessed in children over five years old, in areas where it was previously rare (KM verbal communication).

With regard to vaccine development, immunological and clinical data from large Phase III multicentre vaccination trials of the most promising candidate on the market to date (RTS,S), a vaccine which is directed against the pre-erythrocytic stage of *P. falciparum* malaria, were encouraging. However, longer term follow-up indicated these responses were imperfect and short-lived. In the 18 months following vaccination with RTS,S, vaccine efficacy (VE) against clinical malaria in children was 46% (95% confidence interval (CI) 42% to 50%) but only 27% (95% CI 20% to 32%) in infants vaccinated between 6- and 12-weeks old [18,19]. VE waned with time in both groups and VE was more notable at sites with a lower baseline incidence of malaria [20]. Although further results of the long term follow up are expected, in essence, this is the status of malaria vaccine research and the culmination of over 20 years of development and clinical trials.

### Severe malaria

Formerly, severe malaria in African children was considered to comprise two distinct clinical syndromes: cerebral malaria and severe malarial anaemia. This paradigm was supported by clinical, molecular, immunological and genetic studies and, indeed, substantially influenced their experimental design. As a consequence, it was held that most malaria deaths were attributable to cerebral malaria and, thus, were primarily neurological in origin, with a smaller number resulting from severe malarial anaemia, which could be mitigated by an immediate blood transfusion. Over the last three decades substantial research funding has enabled much clearer and detailed clinical phenotyping of severe malaria in African children, its pathophysiology and complications. What has been determined is that severe malaria encompasses a complex syndrome affecting many organs resulting in biochemical and haematological derangements which have many features in common with the pathophysiological derangements complicating children with severe sepsis. Moreover, in-hospital deaths, most occurring within 24 hours of admission, appear to be a consequence of a wide spectrum of pathophysiological determinants. At the clinical level, a key challenge for health services in Africa, owing to the large burden of paediatric admissions with *P. falciparum* malaria infection, is distinguishing those who are at greatest risk of poor outcome, using largely clinical criteria, in order to target parenteral antimalarials and supportive therapies [21]. This is reviewed in the next section.

### Case definitions and defining those at greatest risk

**Figure 2:**
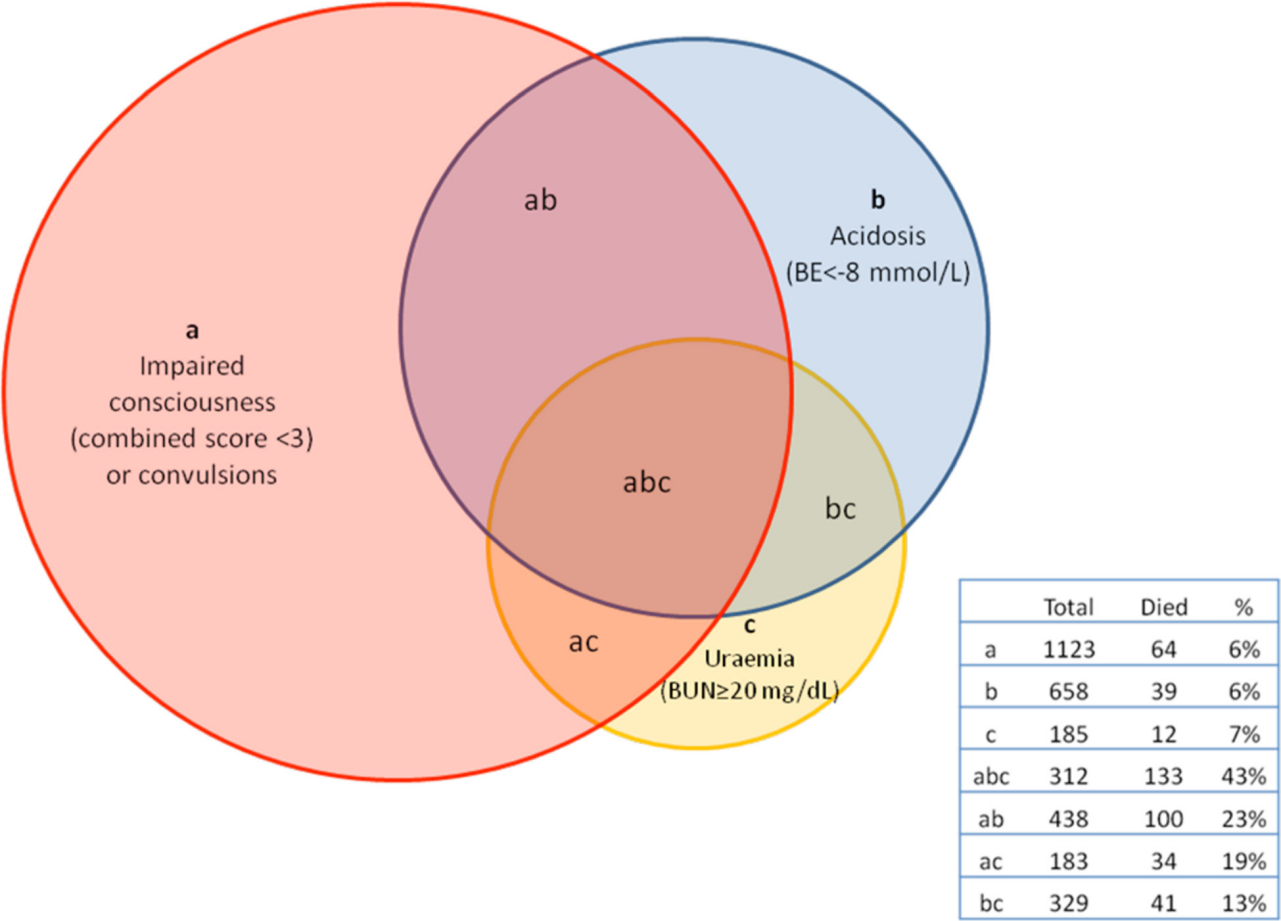
**Combinations of presentations and the associated mortality in children in the AQUAMAT trial.** Venn diagram illustrating the combinations of presentations and associated mortality from von Seidlein *et al.* 2012 [31].

Differentiating ‘true cases’ of severe malaria from other acute infections remains both a clinical and epidemiological challenge, as most symptoms of malaria are indistinguishable from other major causes of morbidity, in malaria-endemic sSA where parasite carriage is the norm rather than the exception. For example, a child with severe respiratory distress and *P. falciparum* malaria could either have severe malaria or very severe pneumonia, an issue that has been covered in a number of publications [22-26]. Determining the cases at greatest risk of poor outcome has been helped by a number of seminal papers. A prospective study of 1,844 paediatric hospital admissions with *P. falciparum* malaria in Kenya established that two clinical features, impaired consciousness (defined as coma or prostration) and respiratory distress (a clinical sign of metabolic acidosis), identified 84% of fatal cases. Prostration (the inability to sit upright in children over eight months or breast feed), any respiratory distress, hypoglycemia, jaundice, or any combination of these, predicted 92% of fatal cases. Relevant to the traditional severe malaria paradigm was that severe malarial anaemia (defined as a haemoglobin (Hb) <5 g/dl) in the absence of these two clinical signs had a very low mortality (1.3%) [27]. Similar findings were reported in Gambian children with severe malaria where neurological status (coma with or without extensor posturing), tachycardia, tachypnoea, hypoglycemia and hyperlactatemia (plasma lactate level, >5 mmol/L) were found as independent indicators of a fatal outcome [28]. Using data from six research units in Africa (SMAC network) the Lambaréné Organ Dysfunction Score was developed, which combines three variables: coma, prostration and deep breathing. The scores ranged from 0 to 3. Sixty-one percent of the children had none of the three signs, 26% had one, 10% had two and 3% had all three signs (case fatality rate 36%). However, it was unclear how the score was constructed since, at an individual level, prostration and coma are mutually exclusive, thus impossible to score 3 [29]. Probably one of the most comprehensive descriptions of the clinical and laboratory complications of severe malaria (and a subsequent evaluation of prognostic markers) comes from the large Phase III randomised clinical trial comparing artesunate and quinine (African Quinine Artesunate Malaria Trial: AQUAMAT) [30]. AQUAMAT included 5,425 participants and was conducted in 11 centres across nine countries, spanning East, West and Central sSA, thus covering a range of malaria transmission intensities. Baseline characteristics included severe acidosis (base excess < −−‐8; 43%), coma (37%), convulsions (32%), severe anaemia (Hb <5 g/dl; 30%), hypoglycaemia (blood sugar level <3 mmol/L; 10%) and compensated shock (9%). In a subsequent sub-analysis of the AQUAMAT data which investigated predictors of poor outcome, four parameters (out of 20 indicators of severity) were identified that were independently associated with fatality in a multivariate analysis [31]. These were base deficit (>8 mmol/L), coma, elevated blood urea nitrogen (BUN, >20 mg/dL) and underlying chronic illness. In those with coma and acidosis, case fatality was 23%, in children with a combination of coma, acidosis and raised BUN, mortality was 43% (Figure 2). Finally, since asymptomatic carriage of parasites is common in endemic areas, a further refinement aimed at improving the specificity of the case definition for either epidemiological or research purposes proposed the addition of a parasitaemia threshold [32]. The malarial-attributable case fraction (MAF) for children with clinical evidence of severe disease increased from 85% to 95% with the inclusion of a parasitaemia threshold of over 2,500c parasites/ml and with the exclusion of those presenting with malaria parasites but had a primary diagnosis of gastroenteritis with severe dehydration, pneumonia, culture-proven bacteraemia, or meningitis.

The latest WHO guidelines [21], informed by systematic reviews by the expert advisory group, have drawn on these resources to inform clinical and therapeutic management recommendations and provided a comparison of the clinical spectrum of malaria in adults and children largely using the AQUAMAT data set [30] and adult data from the corresponding artesunate-quinine trial (SEAQUAMAT) conducted in southeast Asia [33] (Table 1).

**Table 1:** **Comparison of the typical signs and symptoms of severe malaria in South East Asian adults and in African children**

**Sign or symptom**	**Children**	**Adults**
Duration of illness	Short 1–2 days	Longer 5–7 days
Respiratory distress/deep breathing (acidosis)	Common	Common
Convulsions	Common > 30%	Common (12%)
Abnormal Motor Posturing	Common	Uncommon
Prostration/obtundation	Common	Common
Resolution of coma	Early 1–2 days	Longer 2–4 days
Neurological sequelae (following cerebral malaria)	Common (5-30%)	Uncommon (1%)
Jaundice	Uncommon	Common
Hypoglycaemia (blood glucose <2.2 mmol/L)	Common	Less common
Metabolic acidosis (base excess < -8; lactate > 5 mmols/L	Common	Common
Pulmonary oedema	Rare	Uncommon
Renal failure	Rare	Common
CSF opening pressure	Usually raised	Usually normal
Bleeding/clotting disturbances	Rare	Up to 10%
Invasive bacterial infection (co-infection)	Common (10%)	Uncommon (<5%)

### Severe malaria: the clinical syndromes and complications

#### Cerebral malaria

Neurological involvement is common in African children. Typically, children with cerebral malaria present with a history of a febrile illness lasting one to three days with convulsions, impaired consciousness, with or without brain-stem signs. This is strictly defined as an unrousable coma that persists for more than one hour following a seizure (irrespective of anticonvulsant medication) since many children with malaria regain full consciousness after a brief convulsion. The most widely used paediatric classification of impaired consciousness in sSA is the Blantyre Coma Scale (BCS), a practical tool developed for children who are too young to speak [34,35]. Coma is classified as a BSC of 2 (out of a possible 5) or less. The precision of the clinical diagnosis of cerebral malaria, optimal for intervention studies, has been shown to be considerably improved by ophthalmoscopy (in temporarily-dilated pupils) to assess for malaria retinopathy [36]. The observation of a specific malaria retinopathy is supported by autopsy evidence of intracerebral parasite sequestration in a study of 27 Malawian children with fatal cerebral malaria [37].

Neuroimaging data describing the changes associated with cerebral malaria in African children remains very limited. The only prospective study using magnetic resonance imaging (MRI) involved 120 Malawian children with cerebral malaria (strictly defined as a BCS ≤ 2 plus positive retinopathy on ophthalmoscopy) [38]. It identified the most common pathological finding as an abnormal T2 signal in the basal ganglia (84%). In addition, cerebral volume was moderately-severely increased in nearly 50%, supporting evidence from previous autopsy data, which until this study remained inconclusive owing to the possibility that the findings could not exclude post-morbid changes. Potential mechanisms for increased volume suggested including parasite sequestration [37], venous congestion [39] or ischemic (due to seizures) and cytotoxic oedema. The MRI imaging indicated evidence of uncal or cerebellar herniation in nearly 10% [38], an unexpected finding since previous autopsy studies indicated that evidence of herniation was uncommon [37]. Clinically, papilloedema (a putative clinical sign of raised intracranial pressure) is considered uncommon in children with severe malaria. This is supported by a review of ophthalmoscopic findings from 436 children prospectively recruited into several studies of cerebral malaria in Kenya, Malawi and The Gambia [36]. Bilateral swelling of the optic nerve heads occurred in only 6% in marked contrast to the very prevalent retinal changes. Of note, in over 50% of children with papilloedema there was no other abnormality in the fundus; however, prognosis with this sign was very poor.

Seizures are the most common neurological complication of acute *P. falciparum* malaria, manifesting as either simple tonic-clonic or partial convulsive episodes [40] to clinically silent electrical status [41], which may or may not be clinically detected as excess salivation and/or an irregular respiratory pattern [42]. Overall, 38% of Kenyan children had a history of seizures or evidence of seizures at admission to hospital. Multiple seizures occurred in over 50% of these cases, with 22% having a prolonged seizure episode (>30 minutes). The peak prevalence of seizures (49%) occurred in children 27- to 33-months old and were generally associated with a shorter duration of illness and higher *P. falciparum* malaria parasitaemia [43]. Whilst short-lived seizures frequently occur in young children with febrile illnesses, and have limited prognostic significance, whether these are specifically related to acute malarial illness was explored in a paper examining the MAF. Children admitted with two or more seizures in 24 hours prior to admission had very high MAFs, suggesting that acute malaria was the chief cause of convulsions [44].

In addition to seizures, abnormal motor posturing (AMP) is also a common complication of children with cerebral malaria, manifesting as generalised extension of the trunk, increased muscular tone and posturing in the upper and lower limbs and clinically characterised as de-corticate, de-cerebrate or opisthotonic posturing. Whilst the aetiology and pathogenesis remains poorly understood, Idro and colleagues attempted to define the burden and the prognosis of each type of posturing. In 417 Kenyan children with cerebral malaria, 163 (39.1%) had posturing during hospital stay, with 50% developing AMP after admission [45], indicating a higher prevalence than previously described since previous studies mainly reported this complication at the point of admission. De-corticate posturing was the commonest manifestation occurring in 80 (49%), de-cerebrate AMP in 61 (37%) and opisthotonic posturing in 22 patients (14%). Case fatality rate was high in those with AMP (31; 19%), with the terminal mode of death in 19 (61%) suggestive of trans-tentorial herniation. Posturing was associated with age over three years but not with any of the markers of severity, except for opisthotonic posturing which was independently associated with severe metabolic acidosis. Seizures after admission were more common in those with de-cerebrate and opisthotonic manifestations.

Outcome for cerebral malaria remains poor with high in-hospital mortality and neurological sequalae in survivors. In the AQUAMAT trial the subgroup with the greatest mortality were children with coma, with an overall mortality of 359/1,825; 19.6% [30]. Outcome was no better in the artesunate-treated children (18%) than in the quinine-treated children (21%). Estimates of the prevalence of neurological sequelae deficits in survivors of cerebral malaria vary [46-48]. To counter this, a meta-analysis was conducted including studies which had similar case definitions of cerebral malaria, indicating that sequelae occurred in approximately 11% [49]. The most common neurological sequelae reported were ataxia (43%), hemiplegia (39%), speech disorders (39%), blindness (30%), and cognitive [50,51] and behavioural abnormalities [52]. Some of the deficits were transient (for example, ataxia) and fully resolved, whereas others showed improvement over months (for example, hemiparesis) but did not fully resolve [53]. A history of previous seizures, deep coma and focal neurological signs observed during admission were independent risk factors associated with persisting impairments [54].

### Severe anaemia

Severe anaemia in children in sSA remains a leading cause of hospital admission [55] and a major factor in the 800,000 malaria deaths/year [56]. It is a common presenting feature of severe *P. falciparum* malaria in African children, particularly young infants in high transmission settings. It is caused by haemolysis of both infected and uninfected erythrocytes and exacerbated by impaired erythropoiesis [57]. Within international guidelines (WHO) and across different African countries there are some discrepancies with respect to the definition of severe anaemia (and, thus, the threshold haemoglobin or haematocrit level at which transfusion is recommended), some defining severe anaemia as Hb <5 g/dl whereas others use Hb <6 g/dl. For research definitions, severe malarial anaemia is defined as Hb <5 g/dl in recent guidelines (with parasitological evidence of malaria infection) [58].

In terms of prognosis, since the prevalence of pre-morbid chronic mild to moderate anaemia is very common in these populations, presentation in the absence of any other complications has a lower MAF [32] and carries a good prognosis with a case-fatality of <1% [27]. However, if combined with a parasitaemia of ≥2,500 the MAF increases to over 90% [32]. It is also important to note that, in addition to those presenting with severe anaemia, this complication may also develop during hospital admission especially in children with hyperparasitaemia [59], hence the frequent need to monitor Hb. The presence of respiratory distress increases case fatality (approximately 15%) rising to 40% if also complicated by impaired consciousness [27,60]. Although timely transfusion can be life-saving, access to adequate supplies of blood for transfusion remains a key challenge in sSA. In order to preserve this scarce resource, guidelines developed by the WHO encourage the rational use of blood transfusion and recommend that transfusion be reserved for children with profound anaemia (Hb ≤4 g/dL) or for those with a Hb between 4 to 6 g/dL who have additional severity critieria in areas where malaria is hyperendemic but suggest a higher transfusion threshold of 7 g/dl in areas of low malaria transmission [21]. Guideline-adherence remains poor and hampered by the lack of clinical evidence that this policy is safe in both the short and long term. It has been shown that 63% of the early deaths occur in children awaiting transfusion [61-63]. Furthermore, WHO guidelines recommend a standard volume of 20 ml/kg of whole blood (or 10 ml/kg packed cells) for any level below 5 g/dl; however, in practice this results in only a modest Hb rise of mean 2.5 to 3.3 g/dl following initial transfusion with approximately 25% remaining severely anaemic (<5 g/dL) [61]. High case fatalities of children with severe malaria anaemia has resulted in recommendations that the current transfusion guidelines be evidence-based and tested in a clinical trial [64,65].

### Metabolic acidosis and respiratory distress

Improved diagnostic capabilities have led to a better understanding of the importance of metabolic acidosis as a common complication of severe malaria, despite its clinical correlates (deep ‘Kussmaul’ breathing) being recognised for decades [10,19]. Historically, respiratory distress in children with severe malaria has been considered synonymous with congestive cardiac failure [66], although recent work suggests that heart failure is rare, whereas deep breathing or metabolic acidosis [67] is central to the pathophysiology. Moreover, clinical examination findings and chest radiographs are usually normal, providing further evidence that pulmonary oedema or ‘acute respiratory distress syndrome’ is rare in children, although common in adult severe malaria [21].

Despite being a common complication of severe malaria, the aetiology of malarial acidosis is not well understood. At a clinical level metabolic acidosis is more frequent in children with severe anaemia [23], hypovolaemia [21], altered rheological properties of non-parasitised red blood cells (npRBCs) [24] and hepatic or renal dysfunction (due to decreased elimination) [16,25,68]. Both increased production and impaired metabolism of lactate [20] and ketoacids [14,21,22] have been implicated as causes; however, other unidentified organic acids also contribute [21,69,70]. Use of sodium bicarbonate to correct acidosis has fallen out of favour, largely since it failed to address the underlying processes and conferred no clinical benefit [27]. Treatment of acidosis with signs of hypovolaemic shock is covered later in the clinical trials section (see Fluid and vasopressin use for correction of acidosis and shock). Current management guidelines recommend prompt correction of hypoglycaemia, severe anaemia with a blood transfusion, intravenous fluids at 3 to 4 ml/kg/hour (maintenance) and careful monitoring [21].

### Hypoglycaemia

Hypoglycaemia is an important complication of severe *P. falciparum* malaria, especially in children and pregnant women. Ten percent of children enrolled in the AQUAMAT trial had hypoglycaemia (glucose <3 mmol/L) at the point of admission [30] whereas other studies in African children report higher frequencies at and during the course of admission [71-74]. Hypoglycaemia is independently associated with poor outcome [27,53,75], specifically an increased mortality [28,71,76,77], predominantly when accompanied by acidaemia (pH <7.3) or hyperlactataemia (lactate >5 mmol/l) [60,73]. If not carefully monitored, hypoglycaemia can go unnoticed leading to neurological impairment and neurological sequelae [47,54,75]. The aetiology is incompletely understood and is likely to be multifactorial. Depletion of glucose stores due to starvation, parasite utilisation of glucose and cytokine-induced impairment of gluconeogenesis have been implicated [78]. Hyperinsulinaemia, secondary to quinine therapy, has been advanced as an iatrogenic cause and is well established in adults [79,80]. Data on its relationship in African children with severe malaria are relatively few, but what does exist indicates that insulin levels are appropriately low during episodes of hypoglycaemia, either at admission or during quinine treatment [74,81].

In clinical practice there are several definitions of hypoglycaemia, each targeting a different blood glucose threshold. In adults and children hypoglycaemia defined as a blood glucose of <2.2 mmol/L has been shown to be associated with a poor outcome [81] and, hence, this definition is incorporated as the research case definition of severe malaria (for epidemiological and research purposes) [21]. Paediatric treatment guidelines indicate a blood glucose of <3 mmol/L [82], which is now incorporated in the latest 2012 WHO severe malaria guidelines as the threshold for intervention [21]. A retrospective study examining the outcome across the range of blood glucose in 418 Malian children with severe malaria found a 61.5% case fatality at a glucose level of <2.2 mmol/L and 46.2% in those with low glycaemia (blood glucose 2.2 to 4.4 mmol/L) compared to 13.4% in those with normoglycaemia (4.5 to 8.2 mmol/L) [83]. The authors concluded that 6.1 mmol/L is an optimum threshold for intervention, which is substantially higher than currently recommended [58]. The poor outcomes of children at higher glucose levels (up 4.0 mmol/L) were also noted in a general admission population of Kenyan children in whom this was found associated with increased odds of mortality compared to all levels of glucose concentration above this threshold [84]. Risk factors associated with hypoglycaemia (glucose ≤3 mmol/l) at baseline and subsequent development of hypoglycaemia was examined in a retrospective review of 1,234 cases of severe *P. falciparum* malaria [85]. Included in the study were 952 children admitted to hospital before and 282 children admitted after the introduction of the new WHO quinine regimen (when a loading dose of quinine was increased from 10 mg/kg to 20 mg/kg). At baseline (and before use of quinine) hypoglycaemia was present in 16% of the initial cohort and 9% of the second cohort. Following admission (and initiation of intravenous quinine therapy), 15% developed one or more episodes of hypoglycaemia, with over 70% of episodes occurring within 24 hours of admission. There was no evidence of a relationship between quinine dose and any degree of hypoglycaemia after admission. Admission characteristics that predicted later hypoglycaemia were hypoglycaemia, severe anaemia and temperature gradient (a marker of impaired perfusion). Hypoglycaemic episodes were more common during transfusions or periods of disruption of intravenous glucose infusion. Overall, fatal outcome was more common in children developing hypoglycaemia after admission (case fatality 24%) than their euglycaemic counterparts (8%) [85].

As in sepsis, dysregulation of the cortisol, insulin, mitochondrial, or other metabolic pathways have been implicated in the aetiology of hypoglycaemia and deserve further investigation. The evidence supporting the current threshold suggests that intervention at a higher cutoff maybe warranted but this needs to be tested in an adequately powered clinical trial directly investigating the optimum threshold for treating hypoglycaemia and the best mode of treatment (dextrose loading or infusion).

### Evidence for renal involvement in African paediatric severe malaria

Renal involvement in severe malaria is common in adult non-immune case series and semi-immune cases in south- and southeast Asia (around 25% to 50% across various series). Patients may progress to acute kidney injury with anuric or oliguic renal failure [86]. However, in paediatric severe malaria acute renal impairment has been assumed to be minimal [87], but the piecemeal accumulation of data now suggests this problem has been under-recognised. Studies examining prognostic risk factors in Gambian children with severe malaria noted that biochemical evidence of hepatic and renal dysfunction were important additional markers of a poor prognosis, but, contrary to the adult experience in severe malaria, none developed acute renal failure [28]. Several large clinical studies have confirmed the independent significance of raised BUN (>20 mg/dl) or creatinine (>80 mmols/L) and acidosis as poor prognostic signs in severe malaria in African children [31,60]. BUN and pH are measures of hypovolaemia or dehydration but also reflect decreased elimination due to impaired renal function. These measures are key laboratory parameters incorporated in the Risk Injury Failure Loss End-Stage (RIFLE) classification of acute kidney injury [88]. A prospective study in Kenyan children [60] showed that an elevated creatinine (>80 mmols/L) complicated 96/469 (20%) cases at admission and was associated with a case fatality of 26%. In the same study, four factors were independently associated with fatal outcome (deep ‘acidotic’ breathing, hypoxia (SaO_2_ < 90%), hypoglycaemia (<2.5 mmol/L) and creatinine >80 mmol/L (the odds ratio for mortality with elevated creatinine was 5.76 (95% CI 2.3 to 14.3) *P* = 0.0002). In the AQUAMAT trial 1,009/4,148 (24%) had a BUN >20 mg/dL [30] and this parameter was established as one of the three independent clinical features predicting fatal outcome, together with acidosis and impaired consciousness/convulsions [31]. The mortality in children with all three predictors was 43% (Figure 2). In the FEAST trial BUN >20 mg/dL was present on admission in 444/2,118 (21%), with prevalence varying across the sites, being more frequent in hyperendemic areas of Eastern Uganda 265/1,037 (25.6%) and less frequent in Kilifi, Kenya where malaria is mesoendemic (25/189 (13.2%) [89] (KM, unpublished data).

The aetiology of renal injury in the paediatric kidney is unknown and may reflect multiple insults including hypovolaemia or dehydration (pre-renal failure) and direct tubular or glomerular injury. Pathological studies in adults with severe malaria have shown sequestration of parasitised red cells in glomerular and tubulo-interstitial vessels and acute tubular damage due to accumulation of monocytes in glomerular capillaries. The level of sequestration in the kidney was greater in those who developed renal failure than in fatal cases without renal failure [90]. No equivalent autopsy data are available for African children. WHO malaria treatment guidelines consider acute kidney injury as a serum creatinine level >250 mmol/L in adults [91] but does not indicate any criteria for children. Guidelines for the diagnosis and treatment of renal involvement are needed, as is rational integrated fluid management and other supportive treatment in paediatric malaria [87], which do not currently exist.

### Haemoglobinuria

Haemoglobinuria, or blackwater fever (BWF), a syndrome comprising acute intravascular haemolysis, fever and the passage of dark- or red-coloured urine, in patients with current or recent malaria infection, is recognised as a potential complication of malaria. Classically, it was noted to be a consequence of long term use of quinine in non-immune expatriates [92-94]. In African children BWF is considered as a rare complication of malaria [27,28,91,95], which is now supported by data from the AQUAMAT trial [30]. BWF was reported in only 237/5,426 (4.4%) with a case fatality rate similar to the rest of the trial participants (22 deaths: 9.3%) [31]. The development of haemoglobinuria in children after admission, irrespective of treatment arm, was also rare, <1%. A prospective study of severe malaria in children in Papua New Guinea, reported the presence of BWF, attributable to the passage of haemoglobin and/or myoglobin (suggesting muscle breakdown) in the urine, but found no association with glucose-6-phosphate dehydrogenase (G6PD) deficiency [96]. In Vietnam, in a case series of semi-immune adults with BWF, in which only 32% had concurrent *P. falciparum* malaria infection, aetiological factors included quinine treatment (56%) and G6PD deficiency (54%) [97]. In Africa, recent publications describing case-series of BWF tend to be from regions where children are exposed to intense *P. falciparum* malaria transmission (Nigeria, Democratic Republic of Congo (DRC), and Uganda) [98-100]. In a case-control study conducted in the DRC, the majority (88.4%) of BWF cases occurred in the rainy season when BWF was apparently more likely to be related to quinine pre-treatment. In this series, seven children (16.2%) developed acute renal failure [98]. In Nigerian children, a study of severe malaria reported that haemoglobinuria (defined by dip-stick testing of urine) occurred in 48/251 (19.1%), was commonly associated with clinical jaundice (28/48, 58.3%) and that 6% developed renal failure [100]. Unlike the G6PD variants in southeast Asia the African variant (A-) is a mild variant, with most males retaining 20% of normal G6PD activity, sufficient to protect carriers from side-effects of oxidizing drugs or food products. G6PD deficiency is, therefore, unlikely to be a major cause of BWF; if it were, then one would expect that BWF would be more widely reported as a common complication of malaria owing to the high population frequencies in sSA, where it is present in 18% to 20% in males and 5% in females [101].

There is, however, renewed interest in this topic since there have been a number of case series reported from Europe of delayed haemolytic anaemia following treatment of severe malaria with injectable artesunate [102,103]. All cases were successfully treated with transfusions. The syndrome, now labeled as Post-Artesunate Non-infectious Delayed Haemolysis and Anaemia (PANDHA) or PADH for short, is reported to occur in up to 25% of artesunate-treated non-immune adults, typically two weeks after the treatment course. An expert group review panel reviewed world literature and found that PADH was not specific to a particular preparation or manufacturer [104]. Further insights into its pathogenesis have now been established as the delayed clearance (through haemolysis) of once-infected red cells. These ‘pitted’ erythrocytes had been previously spared by artesunate (with quinine treatment they would be removed from the circulation), since the artemisinins induce splenic removal of parasites leaving the red cell intact (a process known as pitting) [105]. Why some patients are susceptible to PADH remains unknown. Data from malaria endemic areas on the occurrence of this condition are lacking.

### Increased risk of bacterial co-infection

Bacteraemia is an important complication of severe malaria in African children but is often under recognised, largely a result of limited and poorly maintained microbiology services across sSA. Nevertheless, data have slowly emerged, mainly from hospitals linked to research programmes, indicating that there may be a biological link between malaria and susceptibility to invasive bacterial infection (IBI). A study in Teule, Tanzania, reported that up to a third of paediatric severe malaria deaths were attributed to bacteraemia [106]. Another large epidemiological study conducted in Kilifi, Kenya, showed that more than 50% of all cases of bacteraemia occurred in the presence of malaria [107]. A systematic review compiling data from epidemiological studies and clinical studies of paediatric hospital admissions of *P. falciparum* malaria (describing IBI) across sSA examined the evidence supporting a biological or epidemiological relationship [108]. In the meta- analysis of ten studies which included data from 15 centers in 11 sSA countries involving 7,208 children with severe malaria, reported over the period 1992 to 2010, found a mean prevalence of IBI co-infection of 6.4% (95% CI 5.81 to 6.98%). Bacterial co-infection resulted in higher case fatalities compared to children with severe malaria alone 81/336 (24.1%) versus 585/5,760 (10.2%). The major pathogens associated with IBI were non-typhoidal salmonellae (NTS), *Escherichia coli* and other enteric gram-negative organisms. There was also some evidence indicating greater risk of NTS IBI in children with malarial anemia [108].

In order to reduce IBI-associated malaria mortality, whilst minimising the risks of excess antibiotic prescribing, further research is needed to establish children at greatest risk of bacteraemia, to inform a policy for targeted antibiotic therapy. For example, Nadjm and colleagues proposed simple clinical criteria [106] which identified 85% of malarial cases with culture-proven bacteraemia. The criteria included an axillary temperature >38°C or <36°C with parasitological evidence of current or recent *P. falciparum* malaria infection plus one or more of: prostration, respiratory distress (chest indrawing or deep breathing), severe anaemia (Hb <5 g/dL), or HIV infection. A pathogen was isolated from aerobic blood cultures in approximately 20% of children (1-week to 12-years old) meeting these criteria. The ‘Teule criteria' are yet to be tested in prospective intervention trials, which could help with future policy of directed (where microbiological services are not available) or short-course empiric therapy with early termination guided by culture results to prevent development of resistance, which is commonly practiced in high-income countries [109].

### Treatment of severe malaria

Quinine has been the mainstay of treatment for African children with severe malaria for decades; it remains effective and little drug resistance has been reported from the continent. The arrival of the artemesinin compounds led to a series of small clinical trials to test their safety and efficacy. The first comparative clinical trials investigated artemether (derivative of dihydroartemisinin, the active antimalarial agent), which because of its poor water solubility can only be injected by the intramuscular route. In clinical trials it proved to be safe but when the data on efficacy were collated in a meta-analysis (including 1,919 randomised patients) it did not lead to an improved overall survival, specifically in African children. This was followed by trials investigating another artemisinin formulation, artesunate, which in contrast to parenteral artemether, is water-soluble and can be given intravenously and is instantly bioavailable [110]. Even intramuscular artesunate is absorbed reliably and rapidly, with peak concentrations occurring within one hour [111]. The SEAQUAMAT trial, conducted in South East Asia (including 202 children) was stopped early after enrolment of 1,461 patients owing to a substantial benefit for patients receiving artesunate [33]. Mortality in artesunate recipients was 15% (107 of 730) compared with 22% (164 of 731) in quinine recipients; a relative reduction of 34.7% (95% CI 18.5 to 47.6%; *P* = 0.0002).

A further trial in African children was justified since expert review at that time considered that the issue of parasite drug resistance to quinine was not an issue (as it was in South East Asia), that quinine remained the optimal antimalarial and owing to the rapid tempo of severe disease in African children with severe malaria compared with adults in South East Asia there were questions about the generalisabilty of the SEAQUAMAT trial. In severe malaria in children most deaths occur within the first 24 hours whereas survival benefit for many patients in the SEAQUAMAT trial occurred after this time point. These issues highlight the importance of equipoise as a strong scientific justification and a prerequisite to conduct further clinical trials with meaningful clinically relevant endpoints (that is, disability-free survival). The AQUAMAT trial enrolled children at the point of hospital admission. The criteria for enrolment were broad and pragmatic with a view to future generalisability of the trial results, principally if the clinician considered the child required parenteral antimalarial medication they were eligible for inclusion in the trial. Diagnosis was reinforced by quality controlled laboratory evidence of *P. falciparum* malaria infection. The primary endpoint was in-hospital mortality. By intention to treat, 297 children (10.9%) receiving quinine treatment died compared to 230 (8.5%) children who received artesunate. Overall, this translated to a relative reduction in mortality of 22.5% (95% CI 8.1 to 36.9) in children in the artesunate arm (*P* = 0.002) [30], with no difference in outcome whether the drugs were administered intravenously or intramuscularly. In the analysis of secondary endpoints there were no differences in outcome across any of the major clinical spectra defining severe malaria. The development of coma and/or convulsions was more common during hospital admission in the quinine arm (*P* = 0.02); but there were no other differences between the arms in number of other complications, additional treatments prescribed, time to recovery or numbers with neurological sequelae – which included 61 persistent mild, moderate or severe such sequelae among survivors [30]. The trial led to a major guideline change for anti-malarial treatment of paediatric severe malaria, which now recommends a preference for parenteral artesunate over quinine [21].

### Adjunctive therapies in severe malaria

The outcome of severe malaria is largely determined by the complications and number of vital organ dysfunction and quality of care available. For most African children this will not include an admission to an intensive care or high-dependency care unit, instead management will be more usually on a busy paediatric ward. It is important to emphasize that the most important factor for management is the implementation of specific antimalarial drug treatment and that this is given as early as possible to reduce the risk of adverse outcome. As is the case in management of severe sepsis, supportive therapies aimed at correcting deranged physiology are also fundamental to improving outcome; however, of the adjunctive agents tested in clinical trials in both adults and children all have failed to show benefit, with the exception of renal replacement therapy in adults [112]. With respect to the high risk group of cerebral malaria, of the 33 clinical trials summarised in Table 2 investigating a range of specific or supportive therapies, 15 have been targeted to the sub-group with cerebral malaria and most have shown harm in those receiving the interventional therapy. These will be reviewed, initially in chronological order, but are largely clustered within a treatment-specific paradigm.

**Table 2:** **Randomised clinical trials of adjunctive therapies in severe malaria in adults and children**

**Study**	**Country**	**Age group**	**Inclusion criteria/ sample size**	**Interventions and dosage**	**Clinical outcome or measures**	**Result**	**Comment**
**Steroids**							
Warrell *et al.* [114]	Thailand	6 to 60 years	Cerebral malaria N = 100	RCT (allocation in pairs) Dexamethasone (DMO) over 48 hours adults 0.5 mg/kg initially then 7 doses of 10 mg each; children 0.6 mg/kg initially then 7 doses (total dose 2 mg/kg) (50) vs placebo (50) plus quinine	1^0^ Death in hospital	Deaths in DMO 8/50 (16%) vs 9/50 (18%) placebo	No benefit. Prolonged coma among the survivors: the interval between the start of treatment and the full recovery of consciousness was 63.2 +/-5.9 hours (mean +/S.E.M.) in the dexamethasone group, as compared with 47.4 +/-3.2 hours in the placebo group (*P* = 0.02). Complications, including pneumonia and gastrointestinal bleeding, occurred in 26 patients given dexamethasone and 11 given placebo (*P* = 0.004)
					2^0^ Neurological sequelae at discharge; time to become rousable and to regain full consciousness; complications		
Hoffman *et al*. [115]	Indonesia	1.5 to 42 years	Cerebral malaria N = 43	Dexamethasone (DMO) (3 mg doses 8 hourly for 48 hours) vs placebo plus quinine	1^0^ Death in hospital	Death DMO: 6/21 (28%) vs placebo 7/22 (31.8%) mean time to coma resolution DMO 83.4 hours (SD = 49.3) vs 80.0 hours (SD 59.4) in placebo	No benefit from addition of DMO in cerebral malaria
					2^0^ Time to become rousable; time to regain consciousness; duration of fever; complications		Coma or hyperparasitaemia (>5%) and hypoglycaemia at any time during hospitalisation were significantly correlated with a fatal outcome, but were not improved by using dexamethasone. DMO arm increased risk of GI bleeding.
**Anti-** **inflamatory: Pentoxifyllin (Pt) and asprin**							
Di Perri *et al.* [117]	Burundi	<14 years	Cerebral malaria N = 100	RCT	Only coma resolution pre-specified (sample size calculation)	Deaths in Pt 0/26 vs 5/30 (17%) control	Stopped after enrolling 56 patients due to civil conflict
				Pentoxifyllin (Pt) (10 mg/kg/day in saline as a continuous infusion over 72 hours) vs control plus quinine		Coma resolution 6 hours vs 4 hours (no SD given). Neurological sequelae Pt arm 2/26 (7.7) vs 2/25 (8%)	
Looareesuwan, *et al.* [118]	Bangkok Hospital for Tropical Diseases	>16 years old	Severe malaria N = 45 (15 per arm)	Three arm placebo controlled trial	Outcomes included mortality, resolution of coma, renal failure, respiration failure, fever and parasite clearance time	No deaths in any group; no difference in coma resolution, duration of intubation, haemodialysis treatments and use of transfusion. Mean parasite clearance times were 59.2, 60.8, and 57.6 hours in Groups I, II, and III	No significant differences among the three treatment groups were found for any of the outcomes. Pentoxifylline as an adjunctive treatment produced no clinically evident benefit.
				I/ high dose of Pt (1.67 mg/ kg/hr)			
				II/ Low dose Pt (0.83 mg/kg/hr).			
				III/ placebo (0.9% NaCl, 1 ml/kg/hr) over 72 hours			
Hemmer *et al.* [119]	Hamburg, Germany	Adults	Falciparum malaria N = 100 projected	Placebo controlled trial Pentoxyfylin (20 mg/kg/ daily infused over 24 hours) vs placebo (saline infusion). One patient in Pt arm did not receive the drug. Only six patients in each group had severe disease (received quinine)	Only endpoint specified was TNFα	No difference in TNFα in Pt arm vs placebo. Subgroup patients with mild disease TNFα levels on day 4 vs placebo (*P* <0.01)	Trial stopped early for futility and concern that there were excess adverse events in the Pt arm.
			52 randomised (27 Pt vs 24 placebo)	Non severe patients mefloquine (Pt 20 mg/kg x 3 doses taken 6 hours apart) or halofantrine (20 mg/kg x 3 doses 6 hours apart)		No effect of time to fever defervescence, parasite clearance or hospital stay	Pt arm AEs included nausea (8), vomiting (7), general uneasiness (2), palpitations (1), and discomfort with intravenous cannulae plastic needle (1) vs 3 in the placebo group: discomfort with intravenous cannulae plastic needle (3) and general uneasiness (1). (*P* <0.05)
Das *et al.* [120]	Orissa, India	Adults	Cerebral malaria N = 52 (30 control vs 22 Pt)	RCT	Not pre-specified	Coma resolution time better in Pt than control 21.6 ± 13.9 vs 63.5 ± 19.7 hours (*P* <0.001)	Pt arm TNFα levels decreased on day 3 (TNF. 47.92 pg/ml SD . ± 27.9; *P =* 0.0029), compared to admission values but in control there was a rise in TNFα levels (TNF . 589 pg/ml SD . ± 602.3; *P* >0.05).
				Group 1 quinine only, Group 2: Pt support (10 mg/kg/day) for the initial 3 days plus iv quinine Method of allocation not specified		Mortality Pt 10% (n = 2) vs 27% (8) in control (*P* >0.05)	
Lell *et al.* [121]	Kilifi, Kenya	9 months to 8 years	Cerebral malaria Estimated N = 20/15	Step-dose escalation	1^0^ Mortality and 3 months neurological sequelae (NS), SAE	Recruited 10 Pt and 5 control	Stopped early for safety concerns, mortality rate was unexpectedly high in the PTX group but sample size too small for definitive conclusions.
				10 mg/kg up to 40 ml/kg Pentoxifyllin vs control	2^0^ coma resolution; fever and parasite clearance	1^0^ Death Pt 4 (40%) vs control: 1 (10%) 2 other SAE in Pt arm 72 hour neurological impairment Pt = 1(17%) vs 2 (50%) NS at 3 months Pt = 0 (0%) vs 1 (25%)	
						2^0^ Coma resolution time (hours) Pt = 8 (4 to 36) vs 8 (4 to 12)	
Hemmer *et al.* (1997) [119]	Hamburg, Germany	>14 years	Severe and non severe malaria (25 had altered or abnormal renal, liver or coagulation tests) N = 97	Prospective RCT	Not pre-specified	Fever defervescence (days)	There were no significant differences in any of the parameters (parasite clearance, defervescence time, or length of hospital stay) between patients receiving heparin, ASA, or the controls.
				3 arms: i/33 low-dose heparin (70 units/kg tds for 5 days subcut, ii/31 iv aspirin (500 mg on day 0, 2 and 4) iv, iii/ 33 control.		5 (range 2 to 9) heparin vs 4 (2 to 9) aspirin vs 3 (2 to 6) control	
						Length of hospital stay 9 (4 to 54) vs 8 (4 to 27) vs 8 (4 to 31) days respectively	
**Monoclonal antibodies, immunoglobulin and anti**-**sequestration therapies**							
van Hensbroek *et al.* [122]	Banjul, The Gambia	1 to 9 years	Cerebral malaria N = 610 (of 624: 14 either died before or unable to receive medication)	RCT Placebo controlled (in a 2 X 2 Factorial trial Monoclonal Ab (Mab: against TNFα) in human serum albumin (0.1%) vs placebo. A single i.v. infusion over 15-mins (other randomization: im artemether vs quinine)	1^0^ mortality in hospital and residual neurologic sequelae (NS) (6 months).	1^0^ Death Mab (60/302; 19.9%) vs (64/308; 20.8%) *P* = 0.9, NS at 6 month 6.8% (15/221) vs 2.2 (5/225) *P* = 0.04	Faster fever clearance on Mab arm. After adjustment for severity features and antimalarial strategy there was no significant survival benefit in children treated with Mab but significantly increased NS rate at 6 month in survivors (15/221 (6.8%) in the Mab arm compared with 5/225 (2.2%) 3.35 (1.08 to 10.4) *P* = 0.02
					2^0^ parasite and fever clearance rates, coma recovery time, neurologic sequelae (NS) at discharge and 1 month	2^0^ NS at discharge 24.4% (59/242) and 22.1% (54/244); *P* = 0 .6 and NS at 1 month 11% (25/228) vs 6.4% (15/234) *P* = 0.1	
Taylor *et al.* [123]	Blantyre, Malawi	1 to 12 years	Cerebral malaria (BCS < = 1) n = 31	Placebo controlled trial i.v. immune globulin (IVIG) (pooled from local blood donations) during the first 3 hours treatment plus quinine	1^0^ Composite of mortality or neurological sequelae	16 received IVIG; 15 placebo	Trial was stopped (based on preplanned stopping rules) – no benefit of IVIG. Placebo vs IVIG: Odds ratio death or sequelae = 0.24 (95% CI 0 to 05, 1 to 26 that is, odds of failing on placebo were about 1/4 of the odds of these events on IVIG
					2^0^ Hours to regain full consciousness; Fever resolution; parasite clearance	Died or sequelae	
						IVIG = 5 + 5 = 10/16 (62.5%) Placebo 1 + 2 = 3/15 (20%)	
Looareesuwan, *et al.* [118]	Bangkok, Thailand	>14 years	Severe malaria N = 23 (12 vs 11)	Placebo controlled trial Polyclonal anti-TNF Fab (n = 12) received 250, 500, or 1,000 units/kg (4 pt each dose). 5 received 2,000 units/kg. Controls (n = 11) saline 100 ml. Treatment allocation not stated.	Clinical endpoints included duration of coma (CM only) development of severe complications. Mechanistic and PK data generated	Coma recovery only reported in 5 patients (3 Fab vs 2 control) Adverse effects: weakness was present longer in controls 5 days (range 3 to 21) vs 4.0 (2 to 14) in Fab arms	Too few patients in the trial with CM (n = 7) to assess efficacy on coma recovery. Only one patient died (control) arm.
Maude *et al*. [126]	Chittagong, Bangladesh	>or = 16 years	*P. falciparum* (>2%) plus modified WHO severe malaria criteria N = 60	Phase II Controlled RCT Levamisole 150 mg po or ng stat dose immediately vs control plus artesunate	Composite: Clinical Death or coma recovery Parasite clearance and lactate Pharmacodynamics Microvascular flow	Death 5/29 (17%) Levamisole vs 9/27 (33%) *P* = 0.22	Levamisole inhibits cytoadherence *in vitro* and reduces sequestration of late-stage parasites.
						No differences in proportions of trophozoites, measures of parasite clearance in blood over 30 hours or effects sequestration	Speculated whether rapid clearance of malaria parasites by artesunate may obscure beneficial effects of levamisole.
**Seizure prophylaxis**							
White *et al.* [127]	Thailand	Children >6 years and adults	Cerebral malaria N = 46	Double blind RCT: 3.5 mg/kg single dose given IM phenobarbitone (Pb) n = 24 Control (n. saline) n = 24	1^0^ Seizure prevention	1^0^ Convulsions in Pb arm 3/24 (12-5%), vs 13/24 (54%) placebo (*P* = 0-006).	Seizures prevention superior
					2^0^ Death	2^0^ Deaths 8 (33%) in Pb arm 5 (20.8) placebo arm (*P* = 0.5)	Small numbers in trial not able to assess effect on mortality.
Kochar *et al.* [130]	Rajasthan, India	Adults age 14 to 74 years	Cerebral malaria N = 185	Randomly assigned:	Not specified	3/102 (3%) developed seizures after admission in Pb arm vs 19/83 (22.9%) control	Reported as a correspondence not formally reported as a peer reviewed manuscript.
				10 mg/kg im one dose of phenobabitone (Pb) (n = 102) Control (n = 83)		Deaths Pb: 29/102 (28.4% vs control 33/83 (39.8%)Pb	
Crawley *et al.* [128]	Kilifi, Kenya	Children 9 months to 13 years	Cerebral malaria N = 340	Placebo controlled trial	1^0^ Seizure prevention	1^0^ Seizure frequency lower in the Pb arm vs placebo group (18 (11%) vs 46 (27%) children had 3 or more seizures OR: 0.32 (95% CI 0.18 to 0.58)	Frequency of respiratory arrest was higher in the phenobarbital arm vs placebo arm
			170 Pb arm/170 placebo arm	A single intramuscular dose of phenobarbital (Pb) (20 mg/kg) or identical placebo plus quinine	2^0^ Death	2^0^ Mortality higher in phenobarbitone arm (30 (18%) vs 14 (8%) deaths; OR 2.39 (1.28–4.64)).	Mortality substantially increased in children who received phenobarbital plus three or more doses of diazepam (OR 31.7 (1.2 to 814))
							Not recommended in CM.
Gwer *et al.* [129]	two centres Kilifi and Kisumu hospitals, Kenya	Children 9 months to 13 years	Blantyre Coma Score < =2 (included CM and non-traumatic encephalopathy)	Placebo controlled trial a single dose of Fosphenytoin or placebo plus quinine (N = 173)	1^0^ Seizure prevention and neurologic sequelae (3 months)	CM sub group (n = 110), Seizure prevalence fosphenytoin (n = 20/54; 37%) vs placebo (n = 21/56; 38%) (*P* = 0.233) Neurological sequelae 5 children (11%) vs 9 (19%) in the placebo arm (*P* = 0.98)	Stopped early (low enrolment/futility). No difference in clinical or EEG evidence of seizures (*P* = .980); gross neurological sequelae (*P* = .283).
				85 received Fosphenytion			Deaths not specifically reported for CM subgroup but overall fosphenytoin 18 (21%) vs placebo 15 (17%) (*P* = .489)
				88 placebo	2^0^ Death		
**Cerebral oedema prevention: osmotherapies**							
Mohanty *et al.* [133]	India	Adults	CM with evidence of brain swelling on admission CT scan	Controlled trial	1^0^ Death	1^0^ Death mannitol 9/30(30%) vs 4/31 (13%) control	Mannitol led to increased mortality (*P* = .11) and prolonged coma duration (*P* = .02)
				1.5 g/kg mannitol followed by 0.5 g/kg every 8 hours vs control (no mannitol)	2^0^ Time to regain consciousness	Time to coma recovery 90 hours (IQR 22 to 380) vs 32 hours (IQR 5 to 168 hours)	
Namutangula *et al.* (2007) [132]	Kampala, Uganda	Children 6 to 60 months	Cerebral malaria N = 156/156	Placebo controlled trial single dose mannitol (1 g/kg) vs placebo plus quinine	1^0^ Time to regain consciousness	1^0^ Time to conscious was18.9 hours (10 to 38) (mannitol) v 20.5 hours (14 to 53) (control)	No difference in any endpoint; no adverse events.
					2^0^ Death	2^0^ Death mannitol 9/75 (12%) vs control 13/76 (16%)	
**Iron chelation therapy**							
Gordeuk *et al.* [135]	Zambia	Children <6 years	Cerebral malaria; unrousable coma N = 78	Placebo controlled Desferrioxamine (DFO) 100 mg/kg/day intravenously for 72 hours vs placebo	1^0^ Mortality	Mortality DFO 7/42 (16.7%) vs placebo 9/41 (22%). Coma recovery DFO: 20.2 hours (N = 41) placebo: 43.1 hours (N = 42) *P* = 0.38	Relative risk of mortality in DFO arm 0.76 (95% CI 0.31, 1.85)
					2^0^ Time to recovery of full consciousness; parasite and fever clearance times		Coma recovery 1.3 times (95% CI 0.7 to 2.3) faster in DFO group than in placebo
Thuma *et al.* [136]	Zambia; two centres	Children <6 years	Cerebral malaria; unrousable coma; clear CSF	Placebo controlled Desferrioxamine (DFO) 100 mg/kg/day intravenously for 72 hours vs placebo N = 352	1^0^ Mortality	Trial stopped early by DSMB after mortality 32/175 (18.1%) DFO arm vs 19/177 (10.7) placebo arm	Increased risk of death in DFO arm (RR 1.70, 95% CI 1.00 to 2.89)
					2^0^ Coma recovery (time to Blantyre Coma Scale: 5); parasite and fever clearance times; parasite clearance day 3	Coma recovery time 1.2 times faster in DFO group vs placebo (*P* = 0.21) DFO: 18.1 hours (N = 143) placebo: 19.0 hours (N = 158) 95% CI 0.97 to 1.6	Persistent seizures >3 lower in DFO 93/168 vs 115/166 (RR 0.8; 0.67 to 0.95) but recurrent hypoglycaemia higher 43/172 vs 29/172 (RR 1.48 (0.97 to 2.26))
Mohanty *et al.* [137]	Mumbai India	13 to 84 years	Severe malaria N = 45	Blinded placebo controlled trial 1. Deferiprone (75 mg/kg/day divided in 2 daily doses) 2. Placebo capsules plus antimalarials (10 days)	1^0^ Mortality	No deaths	
					2^0^ Coma recovery; parasite clearance time	Coma recovery time better in DFO arm 29 hours (SD10) vs 56 (14).	
**Acidosis correction**							
N-acetylcysteine (NAC)							
Watt *et al.* [139]	Bangkok, Thailand	Males 18 to 50 years	Severe malaria N = 30 (15 in each arm)	Phase II placebo controlled trial 1/ NAC dose 150 mg/kg over 15 mins, followed by 50 mg/kg over 4 hours, then 100 mg/kg over 16 hours (in 5% dextrose infusion): vs 5% dextrose (placebo) plus quinine	Lactate acidosis resolution	NAC lactate resolution at 24 hours 10/15 better than placebo (3/15) *P* = 0.011	Small phase II trial
						Median coma resolution faster NAC 24 hours (6 to 60) vs 36 hours (18 to 120) *P* = 0.19.	Overall low mortality and few received renal dialysis.
Treeprasertsuk *et al.* [140]	Thailand	13 years or older	WHO (2000) definition of severe malaria Patient had to agree to stay in-hospital until Day 28 (N = 108; 54 NAC regimens; 54 placebo)	Placebo controlled trial of 3 NAC dosage regimens plus artesunate	Not specified; method of randomization not specified either	54 received NAC Gp 1 (n = 31), Gp 2 (n = 5)^b^ and 18 in Gp 3 (N = 18) Gp 4 (n = 54)	^b^Group 2 oral administration not tolerated as given too early and in a large amount of liquid volume)
				Group (Gp 1) iv: 140 mg/kg loading dose then 70 mg/kg 4 hourly x 18 doses; Gp 2) a single iv loading dose followed by *oral* NAC (see doses Gp 1) Gp 3) iv: loading dose (140 mg/kg) then 70 mg/kg 4hourly for 24 hours then oral NAC Gp 4) placebo		Deaths-only 2 in Group 1. Fever clearance and parasite clearance times – no major differences	Data on fever and parasite clearance time were summarised in table by arm but not compared statistically.
						Withdrawal rate	Inconclusive study; incomplete reporting.
						Gp 1 (4; 13%), Gp 2 (3; 60%), Gp 4 (10; 18.5%)	
Charunwatthana *et al*. [141]	Mae Sot General Hospital, Thailand and Chittagong, Bangladesh	>16 years	Modified WHO severe malaria (N = 108: 56 NAC,52 placebo)	Placebo controlled RCT NAC: 150 mg/kg over 15 mins, followed by 50 mg/kg over 4 hours, then 100 mg/kg over 16 hours (in 5% dextrose) vs saline/5% dextrose (placebo) plus iv artesunate	1^0^ Lactate clearance time, coma recovery time and parasite clearance time. Others: fever clearance time, mortality, red cell deformability	No difference in lactate clearance (adjusted for admission parasitaemia and bilirubin) (hazard ratio 0.98 (0.60, 1.62) or median coma recovery time (72 hours vs 96 hours) adjusted for censored deaths	No difference in case fatality rate: 21 (38%) in the NAC group vs 17 (33%) in the placebo group.
							Treatment with Nacetylcysteine had no effect on outcome in patients with severe falciparum malaria
**L**-**arginine**							
Yeo *et al.* [142]	Timika, Papua, Indonesia	18 to 60 years	Modified WHO severe malaria criteria N = 8	Placebo controlled RCT 12 g L-arginine hydrochloride vs saline over 8 hours plus iv astersunate	Measures of haemodynamic function, endothelial function and nitric oxide bioavailability, lactate clearance	No deaths; no adverse events; arginine did not improve lactate clearance nor endothelial nitric oxide (NO) bioavailability	L-arginine was given to an increase endothelial nitric oxide: since low NO and hypo-arginaemia associated with severe malaria. Study stopped early due to local political instability.
			(6 L-‐arginine; 2 placebo)				L-Arginine was found to be safe but no benefit shown in small number studied. Further studies may require higher dosing.
**Shock management: resuscitation fluids and vasopressors**							
Day *et al.* [68]	Ho Chi Minh City, Viet Nam	Adults	23 patients critically-ill with severe sepsis (n = 10) or severe malaria (n = 13).	Open, randomised, crossover study comparing increasing iv adrenaline doses of 0.1 to 0.5 g/kg per min vs dopamine 2.5 to 10 g/kg per min.	Comparing adrenaline and dopamine effect on acid-base balance and haemodynamics: incidence of drug- associated hyperlactataemia (rise in plasma lactate of >3 mmol/L)	9 received adrenaline first, and 14 received dopamine. Overall, 4 patients required both dopamine and adrenaline to normalise SBP. The full dopamine dose-profile protocol was effected in 19 patients. Development of lactic acidosis curtailed the adrenaline dose-profile at some stage in 16 patients (84%) *P* = 0.0002.	Infusion of inotropic doses of adrenaline in severe infections resulted in the development of lactic acidosis.
							No significant differences were found between sepsis and malaria subgroups with respect to disease effects or responses to treatments.
Maitland *et al.* [60]	Kenya	Children >6 months	Severe malaria plus deep breathing and base deficit >8 N = 53	Dose finding study 0.9% saline (N = 20); 4.5% human albumin solution (HAS (N = 32). Boluses of 10 to 40 ml/kg given over 1st hour after admission	Aliquots of 10 ml/kg given to achieve CVP 5 to 8 cm and improvement in haemodynamic indices. Resolution of acidosis/base deficit reduction at 8 hours	Mean central venous pressure (SE) at admission was 2.9 cm H2O (0.5 cm H2O); in those with base deficit >15 (445 had hypotension); by 8 hours mean CVP = 7.5 mm HG and evidence of resolution of shock and respiratory distress.	Inadequate allocation concealment and inadequate sequence generation.
							No evidence of adverse effects of fluid overload; there were only 4 deaths (case fatality 7.5%) and in the survivors, there were no apparent neurologic deficits at discharge.
Maitland *et al*. [145]	Kenya	Children >6 months	Severe malaria (Hb > 5 g/dl) plus deep breathing and base deficit >8 N = 150	RCT fluid resuscitation (20 ml/kg = bolus) 1. 4.5% albumin N = 61; 2. 0.9% saline N = 56 3. Control^c^ N = 33.	1^0^ Acidosis correction: mean percentage reduction in base excess admission to 8 hours.	1^0^ no difference in the resolution of acidosis between the groups;	^c^ontrol only eligible if base deficit >8 but <15:
					2^0^ Mortality; SAE of fluid overload; neurological sequelae (NS)	2^0^ mortality lower in albumin arm (N = 2 3.6%) vs saline (N = 11, 18%). Relative risk of mortality saline vs albumin 5.5 (95% CI 1.2 to 24.8; *P* = .013)	Subgroup analysis Base deficit >15: Deaths: albumin 2/23 (9%) vs saline 8/26 (33%)
							NS: albumin 3/21 (14%) vs saline 1/18; (6%)
							Base deficit 8 to 15: Deaths: Albumin 0/33, Saline 3/35 (9%) vs Control 2/33 (6%)
Maitland *et al.* [150]	Kenya	Children >6 months	Severe malarial anaemia (Hb < = 5 g/dl) plus deep breathing and base deficit >8; N = 61	Pre-transfusion bolus management RCT	1^0^ Acidosis correction (as above)	1^0^ no difference: albumin group 44% (95% CI 32 to 57%); saline group 36% (16 to 57%); control group 42% (19 to 66%) *P* = 0.7	Tolerability of protocol control 4 (22%) developed decompensated shock: albumin 4 (17%) required emergency interventions; two had salicylate toxicity and 1 had sickle cell anaemia. No need for alternative treatments in the saline group.
				1. 4.5% albumin N = 23	2^0^ Mortality; SAE of fluid overload: pulmonary oedema, neurological events	2^0^ Deaths albumin 4 (17%); saline3 (15%); control3 (17%); No adverse events	
				2. 0.9% saline N = 20	Neurological sequelae		
				3. control N = 18			
Akech *et al.* [151]	Kenya	Children >6 months	Severe malaria, deep breathing and base deficit >8 N = 88	RCT 20 ml/kg over the first hour, repeat if shock persists	1^0^ Acidosis and shock correction	1^0^ No difference in the resolution of shock or acidosis	Per protocol analysis of mortality albumin: 1/40 (2.5%) vs gelofusine 4/40 (10%), (*P* = 0.36)
				1. 4.5% albumin (N = 44)	2^0^ Mortality; SAE related to fluid and neurological sequelae	2^0^ By ITT mortality albumin (1/44; 2.3%) vs Gelofusine (7/44; 16%) *P* = 0.06). No pulmonary oedema/fluid overload events. Fatal neurological events more common in gelatin arm.	
				2. Gelofusin (N = 44)			
Akech *et al.* [152]	Kenya	Children >6 months	Severe malaria (Hb > 5 g/dl) plus deep breathing and base deficit >8 N = 79	RCT 20 ml/kg over the first hour, repeat x1 if shock persists	1^0^ resolution of shock over 8 hours.	1^0^ no difference in 8 hour shock resolution (D70: 23/37 (62%) vs HES: 25/39 (64%), respectively (*P* = .99).	Fluid boluses with either Dextran or 6% HES lead shock and acidosis resolution without eividence of adverse outcome. Specifically, there was no evidence of a renal impairment with HES over 24 hrs of observation, its use was associated with falling creatinine levels and good urine output
				1. Dextran 70 (N = 39)	2^0^ resolution of acidosis, in-hospital mortality, SAEs (allergic reaction, pulmonary oedema, and neurologic sequelae)	2^0^ Acidosis and respiratory distress resolved better in HES: 3/39 (8%) remained acidotic at 8 hours vs D70 10/37 (27%) (*P* = .05). 4 deaths (5%): two per arm. No SAEs	
				2. 6% hydroxyethyl starch (N = 40)			
Maitland *et al.* [89]	Multi- centre 6 sites East Africa	Children	Critically-ill with severe sepsis (N = 1330) or severe malaria (N = 1793) plus shock N = 3121	FEAST trial: an open RCT comparing: 1) 5% albumin bolus g; 2) 0.9% saline bolus (saline- bolus group) 20 to 40 ml/kg over one hour; 3) no bolus (control)	1^0^ 48-hour mortality;	10 48 hour mortality	DMC stopped trial early (after enrollment of 3141 of projected 3600)
		2 months to 12 years		Plus iv antibiotics and/or quinine	2^0^ pulmonary oedema, increased intracranial pressure, mortality or neurologic sequelae at 4 weeks	Sepsis: Bolus 108/884 (12.2%) vs 38/446 (8.5%) RR 1.43 (1.01 to 2.04).	Neurologic sequelae occurred in 2.2%, 1.9%, and 2.0%, respectively groups 1, 2 and 3 (*P* = 0.92), Pulmonary oedema or increased intracranial pressure occurred in 2.6%, 2.2%, and 1.7% (*P* = 0.17), respectively.
						Malaria: 110/1202 (9.2%) vs control 34/591 (5.7%) RR 1.59 (1.10 to 2.31). 20: 28 days mortality 12.2%, 12%, and 8.7% respectively (*P* = 0.004)	
**Transfusion**							
Bojang *et al.* [159]	Banjul, The Gambia	6 months to 9 years	Severe malarial anaemia N = 114 (PCV <15%) Exclusion: immediate transfusion or recent iron treatment	Allocated ‘at random’ to transfusion (15 ml/kg whole blood N = 58) or iron supplementation for month N = 56 (infants = 2.5 mL tds; <20 kg = 5 mL or if > 20 kg = 7.5 mL) 3 times a day plus oral antimalarials	Not specified	Day 7 mean PCV was significantly less in children who received iron than transfused group (*P* = 0.001) Day 28: iron treatment arm had a significantly higher mean PCV than transfused arm	Day 90 not reported as at Day 28, children were allocated randomly to receive weekly chemoprophylaxis with Maloprim (pyrimethamine and dapsone) or placebo
					Clinical reviews (plus malaria slide and haematocrit) on days 7, 28 and 90 after admission.		
Olupot Olupot *et al.* [160]	2 centres, Mbale and Soroti hospitals, Eastern Uganda	2 months to 12 years	Severe anaemia SA (Hb < 6 g/dl) N = 160	Phase II RCT comparing whole blood (30 ml /kg; Tx30: N = 78) vs standard volume (20 ml/kg; Tx20: N = 82)	1^0^ correction of severe anaemia (to haemoglobin >6 g/dl) at 24 hours.	1^0^ Tx30 70 (90%) corrected SA vs Tx20 61 (74%) hazard ratio = 1.54 (95% CI 1.1 to 2.2) *P* = 0.01	Higher volume of blood than currently recommended was safe and resulted in an accelerated haematological recovery in Ugandan children with SA.
			(95 (59%)) had *P falciparum* malaria (by RDT or slide)		2^0^ Re-transfusion; serious adverse events; mortality (48- hour and 28 days; redevelopment of severe anaemia	2^0^ Global Hb increment admission- Day 28 superior in Tx30 (*P* <0.0001); SAE and Death Tx30 = one non-fatal allergic reaction and one death (Tx30) vs 6 deaths in the Tx20 arm (*P* = 0.12)	

AEs, adverse events; CI, confidence interval; CSF, cerebrospinal fluid; CT, computed tomography; CM, cerebral malaria; CVP, central venous pressure; GI, gastrointestinal; Hb, haemoglobin; HES, hydroxyethyl starch; IQR, interquartile range; ITT, intention to treat; N, number; RCT, randomised controlled trial; SAEs= serious adverse events; SBP, systolic blood pressure; SD, standard deviation; S.E.M, standard error of the mean; subcut, subcutaneous; vs, versus.

In general, many of these trials were single-centre phase I or II studies with low sample sizes, often constrained by funding and slow or truncated trial recruitment. A number of trials were terminated early due to political instability, meeting pre-defined safety stopping rule or due to evidence of adverse outcome in the interventional arm. Many trials had primary endpoints targeting clinical surrogates for prognosis (for example, correction of acidosis or cytokine or parasite clearance) and so, as is usual in Phase II trials were not powered to inform meaningful endpoints such as mortality, but are important steps in the justification for further testing in larger trials. Methodological issues were noted in some trials, for example, in some the primary endpoint was not pre-specified, methods of randomisation were not reported and some trials appeared to recruit patient populations with substantially lower overall lower mortality than would be expected, thus reducing the external validity of the results. How we interpret these trials is possibly helped by one overarching emerging theme: that most did not result in a positive outcome for any of the patient-centred endpoints in the interventional arms (that is, those that are clinically relevant). Ordinarily, the early stopping of trials, especially when interim analysis suggests large treatment effects, has been criticised as, more often than not, interim results on small numbers tend to overestimate treatment effects [113] which are often not replicated in larger multi-centre studies. Some therapies may have been disregarded for the same reason in severe malaria; however, it would be difficult to warrant further exploration of a therapeutic intervention in which a trial had been stopped by an external committee reviewing safety for excess harm rather than for futility.

### Steriods: dexamethasone

In order to reduce or prevent cerebral oedema, two separate trials investigated whether the addition of dexamethasone improved the outcome of cerebral malaria in adults and children [114,115]. The trial in Thai adults showed that dexamethasone-treated patients had a more prolonged period of coma (mean 63.2 hours versus 47.4 hours in the placebo arm). Disturbingly, its use led to an increased risk of pneumonia and/or gastrointestinal bleeding in 26/50 compared to 11/50 in the placebo arm [114]. The second trial, conducted in Indonesian children and adults with cerebral malaria, also showed that dexamethasone led to an increased risk of gastrointestinal bleeding with no apparent benefit [115]. A Cochrane review that pooled the data from these two trials (N = 143 participants) found no difference in mortality (relative risk 0.89; 95% CI 0.48 to 1.68; 143 participants, increased risk of GI bleeding in both trials 7/71 (10%) in the intervention-arm compared to 0/72 (0%) in control, a relative risk of 8.17 (1.05, 63.57). The review found no overall increased risk in invasive bacterial infection [116] but highlighted that a major limitation was that neither had trial follow up beyond discharge from hospital, so that the effects of corticosteroids on residual neurological deficits, therefore, could not be assessed. The review concluded there was no evidence to support the use of dexamethasone in cerebral malaria, although numbers involved were small and the assessment of complications in both trials was incomplete.

### Pentoxifyllin

The pathogenesis of severe malaria involves several different processes, including enhanced production of the cytokines including tumour necrosis factor (TNF), sequestration of parasite red cells to the endothelium and decreased erythrocyte deformability. Since pentoxfylline (Pt) has beneficial effects on many of these processes, in particular increasing red cell deformability (and thus improving local tissue perfusion), it was postulated that it might improve outcome in severe malaria. The next phase of trials investigated the addition of Pt to standard antimalarial therapies, primarily aimed at suppression of TNFα production. In total, five trials investigated its use in cerebral malaria, or all-cause severe malaria [117-121]. Two were conducted in African children (Burundi [117] and Kenya [121]). Three trials were stopped early: one due to civil conflict [117] and two due to safety concerns [119,121]. The Burundi trial had enrolled 56 of a projected 100 children with cerebral malaria when it was stopped. There were no deaths in the Pt arm (n = 26) compared to 5 of 30 (17%) in the control arm; 8% of both groups developed neurological sequelae [117]. Owing to the small sample size the results were inconclusive. The second paediatric study, a dose escalation study, conducted in Kenyan children with cerebral malaria was stopped due to safety concerns. A significantly higher mortality occurred in the Pt arm 4/10 (40%) versus 1/5 (20%) in controls [121]. A trial conducted in Thai adults (n = 45) showed no significant differences in any of the arms (which investigated low dose Pt, high dose Pt versus control) for any of the outcome measures [118]. Another trial conducted in Indian adults involving 22 in the Pt arm and 30 controls showed reductions in TNFα (baseline to day 3) in the Pt arm compared to increases in the control arm. Mortality was 25% in the control arm and 10% in the Pt arm [120]. However, there were methodological limitations in this trial since the method for allocation of study arms was not stipulated nor were the study endpoints pre-specified. Owing to lack of overall clinical benefit, and those that were favorable largely related to secondary endpoints, and a suggestion of increased adverse outcome, further exploration of anti-TNF therapies have not been pursued.

### Monoclonal antibodies, immunoglobulins and anti-sequestration therapies

A controlled factorial trial of monoclonal antibodies (Mab) against TNF-α in Gambian children (N = 610) which also compared artemether and quinine, showed faster fever clearance but no survival benefit in children treated with Mab (19.9%) compared to controls (20.8%), after adjustment for antimalarial strategy [122]. The trial also demonstrated a significantly increased risk of long-term (six months) neurological sequelae in survivors 15/221 (6.8%) in Mab recipients compared with 5/225 (2.2%) in controls. The authors suggested that whilst there was evidence that antibody acts to retain and prolong TNF within the circulation (leading to fever reduction) this may inadvertently increase its harmful effects on the vascular endothelium and, in turn, aggravate neurologic complications. A trial investigating pooled intravenous immunoglobulin (IVIG) from blood bank adult donors and, thus, presumptively immune to malaria in Malawian children with cerebral malaria was stopped early after enrolling 31 children for futility (in accordance to a pre-planned stopping rule) [123]. For those receiving IVIG 10/16 (62.5%) died or developed neurological sequelae compared to 3/15 (20%) in the placebo arm. A further placebo controlled trial conducted in 28 Thai adults >14 years old (enrolled using WHO severe malaria criteria) investigated polyclonal anti-TNF-α antibodies in addition to artesunate. Included in the study were seven participants with cerebral malaria, 46% had hyperparasitemia (13 of 28) and 39% had circulatory shock (11 of 28) [124]. Compared to controls antibody treatment reduced IFN-g concentrations in a dose-related manner and unbound TNF-α was undetectable after Fab treatment, irrespective of dose. The composite clinical endpoint, including duration of coma in cerebral malaria and the development of other severe manifestations or complications, was hampered since it was only possible to record coma recovery in five patients; hence, the results were largely inconclusive.

A controlled Phase II trial investigating the use of the oral anthelmintic drug levamisole hydrochloride (previously shown to inhibit cytoadherence *in vitro* and reduce sequestration of late-stage parasites in uncomplicated *P. falciparum* malaria [125]) included 56 Bangladeshi adults with criteria for all-cause severe malaria [126]. Linked pharmokinetic studies indicated absorption of levamisole was reliable with a mean plasma level of 97% of expected levels in healthy adults. However, there was little marked effect upon parasite sequestration, lactate clearance (a putative marker of tissue oxygenation and microcirculatory perfusion) or mortality. The authors suggested that despite previous observations in uncomplicated malaria treated with quinine, rapid parasite clearance with intravenous artesunate may have obscured any marginal benefit of levamisole on parasite sequestration.

### Seizure prophylaxis

Since convulsions are common in severe malaria, in particular the sub-group with cerebral malaria, and associated with adverse outcomes including death and neurological sequelae (in children), routine administration of anti-epileptics to prevent the seizures was suggested. In a double-blind controlled trial in Thailand involving both children and adults with cerebral malaria, randomised to receive either a single dose of phenobarbitone (Pb) or placebo, there was a lower seizure rate in the Pb group at 12.5% (3/24) compared to 54% (13/24) in the placebo arm (*P* = 0.006) [127]. There was no effect on mortality, eight (33%) versus five (21%), respectively. Based on this small study the authors recommended that guidelines should advocate that all patients with cerebral malaria should receive a single intramuscular loading dose of Pb. A large single centre paediatric placebo-controlled trial (N = 340) conducted in Kenyan children with cerebral malaria, designed to provide the evidence for this new recommendation, examined a single intramuscular dose of Pb (20 mg/kg) versus placebo. As expected Pb recipients had a lower rate of seizures (defined as three or more seizures of any duration) than the placebo recipients (18 (11%) versus 46 (27%) odds ratio 0.32 (95% CI 0.18 to 0.58)) but the case fatality rate was more than double in the Pb arm (30 (18%) compared to placebo 14 (8%) deaths, relative risk 2.39 (95% CI 1.28 to 4.64)) [128]. The chief mode of death in those receiving Pb was respiratory arrest. Following this trial the guidelines changed and no longer recommend Pb prophylaxis in cerebral malaria. A further placebo controlled trial, conducted at the same centre in Kenya, in non-traumatic encephalopathy (including a cerebral malaria subgroup (N = 110)) investigated the use of fosphenytoin (a drug often used for seizure prophylaxis in neuro-trauma that has minimal cardiorespiratory side effects). It was terminated due to slow recruitment/futility. At least one seizure (monitored clinically and by electroencephalogram) occurred in 33/83 (40%) in children receiving fosphenytoin versus 32/88 (36%) receiving placebo (*P* = 0.733) [129]. In the cerebral malaria subgroup the prevalence of seizures in the fosphenytoin arm was 20, 37% which was identical to the placebo arm 21, 38%. Overall, 18 children treated with fosphenytoin (21%) died compared with 15 in the placebo arm (17%) (*P* = 0.489). In survivors rates of neurologic sequelae at three months were the same (10%) in both arms [129]. A study from India, reporting results from a clinical trial in adults as a correspondence piece, gave only limited details of the clinical trial methodology. The successful use of prophylactic Pb reported in this trial is questionable since it was not reported in full [130].

### Osmotherapy: mannitol for treatment or prevention of cerebral oedema

Leading up to these trials were two observational studies, one case series and one uncontrolled trial, investigating mannitol and other osmotic diuretics as adjuncts for treating brain swelling (cerebral oedema) in African children with cerebral malaria [39,131]. These early studies suggested that mannitol may improve outcomes [39], and case series of children receiving urea with dexamethasone was reported to have a dramatic improvement [131]. A subsequent placebo-controlled trial, conducted in 156 Ugandan children with cerebral malaria, investigated a single dose of 5 ml/kg of 20% (1 g/kg) of intravenous mannitol infused over 20 minutes in addition to intravenous quinine. No important differences were found in the main outcomes, including time to regain consciousness or death [132]. Six children had postmortems of whom five had signs of anoxia and cerebral oedema; the sixth child had signs of acute tubular necrosis at autopsy. A controlled trial examining the use of 1.5 g/kg mannitol followed by 0.5 g/kg every eight hours in Indian adults with death as the primary endpoint found an increased mortality in those receiving mannitol, 9/30 (30%), compared to 4/31 (13%) in the control (no mannitol) arm [133]. Mannitol also prolonged coma resolution. Whilst there is good evidence that brain swelling complicates both adult and paediatric cerebral malaria, trials to date have shown no benefit of osmotherapy or steroids (see earlier).

### Iron chelation

The use of iron chelating agents was considered for adjunctive therapy in severe malaria. The positive hypothesis suggested that by chelating free iron these drugs would thus withhold iron which is required by malaria parasites to mature and multiply and, thus, limit or inhibit parasite reproduction rate. A Cochrane review of the data on the uses of iron chelation was conducted in 2007 [134]. Whilst seven trials involving 570 participants met the inclusion criteria, four of these were excluded as they included non-severe or asymptomatic malaria or had methodological issues. In children with severe malaria two controlled trials examined desferrioxamine (DFO) [135,136] with cerebral malaria and one studied deferiprone [137] in Indian adults (13 to 84 years) with severe malaria.

The first study conducted in a Zambian hospital involving 83 children with cerebral malaria indicated a lower mortality in DFO recipients, 7/42 (16.7%), compared to placebo, 9/41 (22%). Moreover, time to coma recovery was faster in the DFO arm [135]. A second, larger paediatric trial conducted at two centres in Zambia was stopped early on the recommendations of the data monitoring committee for safety, as there were more deaths in the DFO arm, 18.3% (32/175) compared to 10.7% (19/177) in the placebo group [136]. Overall, the pooled estimate for both trials (435 participants) indicated a non-significant increased risk of death in the DFO group. The risk of experiencing persistent seizures was lower with DFO compared with placebo (RR 0.80, 95% CI 0.67 to 0.95; 334 participants, one trial), but adverse effects (including phlebitis and recurrent hypoglycaemia) were more common with DFO (risk ratio for hypoglycaemia 1.48 (95% CI 0.97 to 2.26)) [134]. The adult trial comparing deferiprone with placebo found that the deferiprone group had significantly faster coma recovery and parasite clearance [137]. No adverse effects were reported for this trial. No evidence of benefit or harm overall was concluded by Cochrane review [134].

### Acidosis correction

N-acetylcysteine (NAC) has been proposed to lead to correction of metabolic acidosis by its antioxidant effect acting through direct scavenging of free radicals and replenishment of glutathione and cysteine. NAC has also been shown to have anti-parasitic effects [138]. Three trials have examined effects of different doses as adjunctive treatments to quinine or artesunate. The first, a small placebo controlled trial of NAC in Thai adults (N = 15 in each arm) had lactate clearance as the primary endpoint. Lactic acidosis resolution was better at 24 hours in the NAC group (10/15) than in the placebo group (3/15) (*P* = 0.011), as was median coma resolution although with wide variation and, thus, was non-significant. Need for renal replacement therapy (two in each arm) or death (arm in each arm) were similar [139]. A placebo controlled trial of three different NAC dosage regimens given in addition to artesunate had some methodological issues since the key endpoints were not pre-specified; methods of randomisation were not indicated and the study required patients to remain in hospital for 28 days. Few differences were found in fever, parasitaemia clearance and adverse medical events, although the reporting of these was incomplete. There were only two deaths in the trial indicating that the patients probably did not have severe malaria [140]. A multisite double-blind placebo controlled trial on the use of high dose intravenous NAC as adjunctive treatment to artesunate in 108 adults in South East Asia found that NAC had no significant effect on mortality, lactate clearance times (*P* = 0.74) or coma recovery times (*P* = 0.46). Parasite clearance time was increased but largely attributable to differences in baseline parasitaemia,nor did NAC have any effect on red cell deformability properties [141]. In summary, despite some methodological concerns and the small size of the trials, the limited data available from these do not provide justification for further investigation of NAC as an adjunctive treatment. Similarly, a lack of efficacy was found in another small study of L-arginine; however, the results were not conclusive since the trial was halted early due to political instability [142].

### Fluid resuscitation, inotrope and and vasopressors: shock and acidosis

Both dopamine and adrenaline (epinephrine) are used in the management of infection-induced haemodynamic shock as second line treatments when shock is refractory to fluid boluses or when hypotension prevails [109]. Dopamine has been used to improve mean arterial pressure (MAP) and cardiac output, primarily by increasing stroke volume and heart rate and low dose therapy has been used in the past to treat pre-renal acute kidney injury. Dopamine however carries an increased risk of supraventricular and ventricular arrhythmias. Adrenaline increases the MAP since it is a powerful vasopressor agent, but increases the risk of aerobic lactate production [109]. In Vietnamese adults with severe sepsis (n = 10) and severe malaria (n = 13), a crossover trial examining increasing intravenous adrenaline infusion compared to increasing dopamine doses found adrenaline and dopamine infusions led to significant mean increases in cardiac index (which was greater with adrenaline than dopamine), increased MAP and a reduction in systemic vascular resistance. However, inotropic doses of adrenaline resulted in the development of lactic acidosis and falling pH and thus curtailed the adrenaline dose-profile at some stage in 16 patients (84%). No significant differences were found between sepsis and malaria subgroups with respect to disease effects or responses to treatments [143]. Further investigation of adrenaline was, therefore, not warranted.

Justification for investigating fluid resuscitation as an adjunctive treatment in children with severe malaria originated from the high mortality noted in association with metabolic acidosis [27,67,144]. It was postulated that in children with severe malaria complicated by metabolic acidosis in accordance with other causes of paedaitric severe life threatening illness this is commonly due to some degree of hypovolaemia [145], although this view was controversial in severe malaria [146]. Prospective studies had identified markers of impaired perfusion (delayed capillary refilling time, weak pulse, subnormal systolic blood pressure and increased creatinine) in children with metabolic acidosis as key correlates with poor outcome [60]. Initial dose-finding studies in this subgroup established that boluses of 20 to 40 ml/kg normalised low central venous pressure, reduced tachycardia and tachypnoea without adverse consequences [147] and further studies established appropriate effects of fluid therapy on myocardial performance [148]. In total, five Phase II trials were conducted in Kenyan children with severe malaria comparing different fluid types on physiological endpoints, including correction of metabolic acidosis (base excess) and haemodynamic parameters as well as mortality and neurological sequelae in survivors. The results of these trials are individually summarised in Table 2 [60,149-152] but have been formally collated (four of five) in a meta-analysis examining types of fluid resuscitation therapy published in 2010 [153]. This review compiled data from all published paediatric clinical trials of fluid resuscitation. In the subgroup with malaria these were the only four trials (none of which had a control arm), and all had similar designs, all showing comparable reductions in acidosis (the primary endpoint) as well as features of shock in children receiving either 0.45% human albumin solution, 0.9% saline or Gelofusine (a gelatin based colloid) [153]. When the mortality data from all four trials were combined in the meta-analysis this indicated that albumin significantly reduced mortality (secondary endpoint) compared to either of the other fluids (saline or Gelofusine), risk ratio 0.31 (95% CI 0.14 to 0.68) (*P* = 0.004). There was a non-significant increase in neurological sequelae in survivors in those treated with albumin 9/97 (9%) compared to other resuscitation fluids, 6/54 (6%) in saline and 1/37 (3%) in gelofusine [153]. A further trial comparing Dextran 70 (D70) (n = 39) and 6% hydroxyethyl starch (HES) (n = 40) found no difference in the primary outcome (shock resolution at eight hours) (D70: 23/37 (62%) versus HES: 25/39 (64%)), although resolution of acidosis was better in the HES arm. Overall, case fatality (four in each arm, 5%) was similar [152].

The Fluid Resuscitation as A Supportive Therapy trial (FEAST) was conducted to provide evidence on the best fluid management for children with shock and severe febrile illness for doctors practicing in hospitals in resource-limited settings [89]. FEAST was a multicenter three-arm controlled trial comparing albumin bolus, and saline bolus to no bolus control. FEAST was conducted at six centres in three East African countries and involved 3,121 children enrolled at the point of hospital admission. Shock was defined pragmatically (incorporating parameters from all the published paediatric shock definitions including modest hypotension) and the controlled part of the trial (FEAST A) only excluded children without severe hypotension. Two major subgroups included in the trial were those with severe sepsis (n = 1,330) and severe *P. falciparum* malaria (n = 1,793). The data monitoring committee stopped the trial early after enrollment of 3,141 of a projected 3,600 due to safety concerns. The primary endpoint, 48-hour mortality, was significantly worse in the two fluid bolus arms, 111/1,050 (10.6%) in the albumin-bolus and 110/1,047 (10.5%) in the saline-bolus, compared to the control (no bolus) 76/1,044 (7.3%); relative risk (95% CI) any bolus versus control 1.45 (1.13 to 1.86, *P* = 0.003). Secondary endpoints including 28-day mortality were worse in the bolus arms, 12% versus 8.7% in the control arm. There was no difference between the treatment arms in the number of adverse events (suspected pulmonary oedema, brain swelling or allergic events). In the subgroup with severe malaria, 48-hour mortality was 110/1,202 (9.2%) in the bolus arms compared to 34/591 (5.7%) in the control (no bolus) arm, a relative risk of death of 1.59 (1.10 to 2.31). In all pre-specified sub-groups analysis, there was no evidence of a benefit of fluid boluses [89]. In a subsequent publication, early shock reversal at one hour (responders), the central principle of the surviving sepsis guidelines, was superior in the bolus arms compared to no bolus control (43% versus 32%, *P* <0.001). Despite this, excess mortality with boluses was still evident in both ‘responders’ (relative risk 1.98 (0.94 to 4.17), *P* = 0.06), as well as ‘non-responders’ at one hour (relative risk 1.67 (1.23 to 2.28), *P* = 0.001), with no evidence of heterogeneity (*P* = 0.68) [154]. Examining the terminal modes of death, the major difference between bolus and control arms was the higher proportion of cardiogenic shock in the bolus arms (n = 123; 4.6% versus 2.6% in controls, *P* = 0.008), rather than respiratory events or neurological terminal clinical events [154]. A major issue raised following the publication of FEAST was whether the criteria used to define shock in the trial were applicable to other guidelines, including the WHO definition of shock. A further publication provided data to address this issue showing that of the relatively limited number of children fulfilling the WHO shock definition 48-hour mortality was 24/50 (48%) in those receiving boluses compared to 3/15 (20%) in the no-bolus control, an increased absolute risk of 28% and a relative increase of 240% (*P* = 0.07 by two-sided Fisher’s exact test). Moderate hypotension, consistent with many definitions in paediatric shock, was also associated with increased 48-hour mortality on boluses (an absolute risk of 9.4% (‐2.6 to 21.4%)) [155].

A systematic review conducted after the FEAST trial concluded that the majority of evidence from randomised trials to date came from the FEAST trial and that boluses significantly increased mortality of children in shock, compared to those who did not receive boluses. The evidence was considered to be of high quality and sufficiently precise [156]. Prior to FEAST the evidence supporting fluid resuscitation was reviewed in the 2008 Surviving Sepsis Campaign Guidelines which was informed by a modified Delphi process, graded the current paediatric recommendation (20 ml/kg boluses over five to ten minutes up to 60 ml/kg) as 2C, indicating a weak recommendation with low quality of evidence [109,157]. The systematic review concluded that withholding of bolus fluids should be considered for populations similar to those enrolled in FEAST and that there remained important questions about the extent to which these results are applicable to other populations [156].

### Transfusion

A Cochrane review including the only two African randomised controlled trials (RCTs) [158,159] conducted before 2000 (involving 114 and 116 children randomised to blood transfusion or oral haematinics) concluded that there was insufficient information on whether routinely giving blood to clinically stable children with severe anaemia either reduces death or results in a higher haematocrit measured at one month, and indicated the need for a definitive trial [64]. Since then a recent trial has evaluated the safety and efficacy of a higher volume of whole blood (30 ml/kg, n = 78) against the standard volume (20 ml/kg, n = 82) in Ugandan children which was found to be safe and resulted in an accelerated hematological recovery in children with severe anaemia [160]. A clinical trial is underway that is evaluating whether a liberal rather than conservative transfusion strategy in terms of a larger initial volume of transfused blood and incorporating a higher threshold of haemoglobin of <6 g/dl for transfusion will decrease both short and long term mortality (TRACT trial: ISRCTN84086586).

## Conclusions

In conclusion, human trials carried out on the basis of pathophysiology studies, have so far made little progress on reducing mortality, despite appearing to reduce morbidity endpoints (such as convulsions, shock reversal or improvements in acid base balance or cytokines); more often than not, the supportive interventions have shown harm. A new line of investigation that aims to disrupt sequestration [161] looks promising and early phase trials are planned. For the key risk factors for poor outcome – coma, abnormal renal function, hypoglycaemia and acidosis – further studies and trials need to be conducted to help us understand how best to treat these complications.
